# Improving Health and Reducing Comorbidity Associated with HIV: The Development of* TAVIE en santé*, a Web-Based Tailored Intervention to Support the Adoption of Health Promoting Behaviors among People Living with HIV

**DOI:** 10.1155/2017/4092304

**Published:** 2017-03-14

**Authors:** José Côté, Sylvie Cossette, Pilar Ramirez-Garcia, Geneviève Rouleau, Patricia Auger, François Boudreau, Marie-Pierre Gagnon

**Affiliations:** ^1^Research Centre of the Centre Hospitalier de l'Université de Montréal, 900 Saint Denis Street, Montréal, QC, Canada H2X 0A9; ^2^Research Chair in Innovative Nursing Practices, 900 Saint Denis Street, Montréal, QC, Canada H2X 0A9; ^3^Faculty of Nursing, Université de Montréal, 2375 Chemin de la Côte-Ste-Catherine, Montréal, QC, Canada H3T 1A8; ^4^Research Center of the Montréal Heart Institute, 5000 Bélanger Street, Montréal, QC, Canada H1T 1C8; ^5^Faculty of Nursing, Université du Québec à Trois-Rivières, 3351 Boul. des Forges, CP 500, Trois-Rivières, QC, Canada G9A 5H7; ^6^Research Centre of the Centre Hospitalier Universitaire de Québec, 2705 Boulevard Laurier, Québec City, QC, Canada G1V 4G2; ^7^Faculty of Nursing Sciences, Université Laval, 1050 Avenue de la Médecine Local 3645, Québec City, QC, Canada G1V 0A6

## Abstract

*Background*. In the domain of health behavior change, the deployment and utilization of information and communications technologies as a way to deliver interventions appear to be promising. This article describes the development of a web-based tailored intervention,* TAVIE en santé*, to support people living with HIV in the adoption of healthy behaviors.* Methods*. This intervention was developed through an Intervention Mapping (IM) framework and is based on the theory of planned behavior.* Results*. Crucial steps of IM are the selection of key determinants of behavior and the selection of useful theory-based intervention methods to change the targeted determinants (active ingredients). The content and the sequence of the intervention are then created based on these parameters.* TAVIE en santé* is composed of 7 interactive web sessions hosted by a virtual nurse. It aims to develop and strengthen skills required for behavior change. Based on an algorithm using individual cognitive data (attitude, perceived behavioral control, and intention), the number of sessions, theory-based intervention methods, and messages contents are tailored to each user.* Conclusion*.* TAVIE en santé* is currently being evaluated. The use of IM allows developing intervention with a systematic approach based on theory, empirical evidence, and clinical and experiential knowledge.

## 1. Introduction

The life expectancy of HIV-infected individuals with access to antiretroviral therapy (ART) is now measured in decades and, among those optimally treated, it can approach that of uninfected populations [1, 2]. However, the long-term use of ART, the normal aging process, and the presence of certain lifestyle risk factors have been associated with onset of metabolic disorders, particularly glucose metabolism dysfunction and dyslipidemia [3–7]. These disorders, as well as other factors, can increase risk for diabetes and cardiovascular disease [8].

The interventions developed in recent years to prevent comorbidity among people living with HIV (PLHIV) have mostly targeted three lifestyle risk factors, namely, tobacco use [9, 10], physical inactivity [11, 12], and poor diet [13]. The literature in this regard is fragmentary. However, two recent literature reviews [9, 10] that examined, respectively, 9 and 10 studies of smoking cessation interventions for HIV-infected adults concluded that such interventions could be effective but that innovative individually tailored interventions needed to be developed for this population. Clearly, case management of PLHIV today must include risk-reducing interventions aimed at supporting PLHIV in their efforts to adopt positive health behaviors, especially quitting smoking, exercising, and developing better eating habits [14].

In the field of health behavior change interventions, the use of information and communications technologies (ICT) as a means of intervention delivery shows tremendous promise and the indubitable potential to promote healthy behaviors [15, 16]. In a systematic review, Hou and colleagues (2014) concluded that web-based interventions produced positive results in terms of behavioral health outcomes [16]. In a meta-analysis of 40 randomized controlled trials (RCT) of web-delivered tailored health behavior change interventions, Lustria and colleagues (2013) reported that such interventions had a significantly greater effect on health behaviors, such as smoking cessation and healthy eating, compared with control conditions (e.g., nontailored intervention or waitlist control group) [15]. A tailored intervention can be defined as a change strategy devised for a specific person based on the person's individual characteristics identified beforehand through an individual evaluation [17–19]. The specificity of tailored interventions allows achieving greater significant results in terms of behavior change, especially if the person receives feedback adapted to the personal characteristics of their health behavior [20–22]. In this regard, the use of ICT seems, in theory, to offer many advantages in a public health perspective [23]. Uppermost among these is the capacity to disseminate a large amount of information formulated for individual needs, in a dynamic and sequential fashion, anywhere, anytime. Much of the user appeal of this approach derives from the possibility it affords of accessing customized information at one's convenience.

In light of the above, we developed a web-based tailored nursing intervention called* TAVIE en santé* to help PLHIV adopt healthy behaviors. The groundwork for the intervention was provided by the earlier creation of a virtual nursing intervention concept called* TAVIE,* the French acronym for* Traitement Assistance Virtuelle Infirmière et Enseignement *(Treatment Virtual Nurse Assistance and Teaching), and of an innovative web platform. The purpose of this paper is to describe how the* TAVIE en santé* intervention was developed.

## 2. Methodology

The web-based tailored nursing intervention was developed using the Intervention Mapping (IM) protocol developed by Bartholomew and colleagues [24, 25]. The strength of the protocol lies in the fact that it integrates theory and empirical evidence to develop an action plan for the primary, secondary, and tertiary levels of intervention. IM is a six-step process. The first step consists in conducting a needs assessment to identify the health problem in the target population and associated determinants. In step two, matrices of change objectives are created to establish the behaviors and their determinants that should be targeted. Step three serves to select the theory-based intervention methods and the practical applications for changing the health behaviors via their determinants. Step four consists in developing the content and sequence of the intervention. Step five involves implementing the intervention and step six involves evaluating it.

Section 3 describes how the IM process was applied in developing the* TAVIE en santé* web-based tailored nursing intervention.

## 3. Results

### 3.1. Step  1: Needs Assessment

A needs assessment was carried out to document the lifestyle risk factors of PLHIV. In this case, determinants associated with the behavior changes sought were identified through screening of relevant literature.

#### 3.1.1. Health Problem: Lifestyle Risk Factor

The selection of the topics of interest was based on clinical relevance and published data. Tobacco smoking has been found to be two to three times as prevalent among PLHIV as in the general population [9, 10, 26–29], which means that 40% to 70% of PLHIV are smokers. Moreover, a literature review by Santos-Lozano and Garatachea (2011) suggested that PLHIV were drastically less active than the healthy population (e.g., as measured by number of steps walked per day and time spent doing physical activity) [30]. Also, a systematic review by Schuelter-Trevisol et al. (2012) revealed that up to 73% of PLHIV were sedentary [31]. Finally, research has shown that PLHIV were more likely to exceed intake recommendations for saturated fat [32, 33] and dietary cholesterol [34].

#### 3.1.2. Predictors of Healthy Behaviors: Theory of Planned Behavior

**Figure 1:**
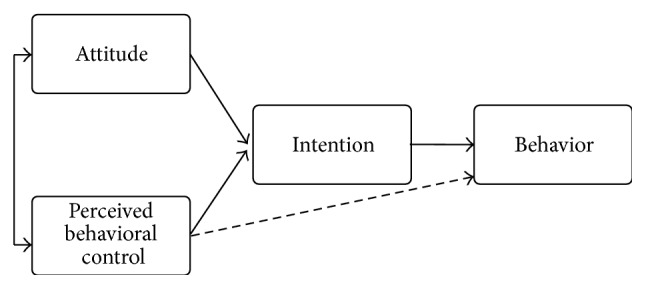
Targeted determinants of behavior in the TAVIE en santé intervention (figure adapted from Ajzen (1991) in Godin (2012) [35]).

The meta-analyses carried out by Armitage and Conner (2001), Godin and Kok (1996), Sheeran (2002), and Webb and Sheeran (2006) suggested the Ajzen's theory of planned behavior was very good for predicting and explaining numerous health behaviors [35–39]. According to this theory, whether a health behavior is adopted is determined by intention (motivation), which in turn is explained by attitude, subjective norm, and perceived behavioral control. Attitude and subjective norm influence intention, which predicts whether the behavior will be adopted. However, perceived behavioral control is a determinant that can influence the behavior directly when the behavior is not under volitional control. Within the framework of the proposed intervention aimed at quitting smoking, engaging in physical activity, or adopting a healthy diet, three determinants of behavior were targeted: intention, attitude, and perceived behavioral control (see Figure 1).

### 3.2. Step  2: Matrix of Change Objectives

The matrix of change objectives is the basic tool used in IM for conceptualizing and developing interventions [24]. The matrix is created by the intersection of performance objectives and determinants (i.e., factors that explain and/or predict the adoption of healthy behaviors). The endpoints, or proximal program objectives, state what must be learned and performed by the target group in order to optimize health behaviors.

The first task in step 2 is to set expected intervention outcomes. The aim of our nursing intervention is the adoption of the following healthy behaviors: smoking cessation, physical activity, or healthy eating. Intervention recipients are asked to choose which of these three behaviors they wish to change. The second task in step 2 is to subdivide behavioral outcomes into performance objectives. These describe what people need to do in order to achieve the desired change: (1) identify the health behaviors to adopt; (2) choose to adopt one of the proposed behaviors and undertake the process of adopting the behavior, begin the behavior, and act accordingly; and (3) engage in and consolidate the behavior, overcome possible barriers by deploying means and reminding oneself of the benefits of adopting the behavior. The third task in step 2 is to select and refine the key determinants of behavior amenable to change.  Table 1 presents the matrix of change objectives.

### 3.3. Step  3: Theory-Based Intervention Methods and Practical Applications

Step  3 in IM consists in selecting useful theory-based intervention methods to change the targeted determinants of behaviors. Practical applications, for their part, are specific techniques or strategies that serve to organize and operationalize the theoretical methods. In our case, three methods are used for all the targeted determinants: tailoring, modeling, and feedback/reinforcement. Specific methods are also used for each determinant. To change attitude, the following methods are employed: behavioral belief selection and persuasive communication. To identify behavioral beliefs, the virtual nurse in the intervention provides information on the benefits of the target behavior. She invites participants to identify the advantages and disadvantages for them of adopting the chosen behavior. She also refers to examples provided by other PLHIV to help with the process. Here is an example of what the virtual nurse might say:

Right now, you have little or no intention of engaging in physical activity and barely see any advantage to doing this. Tell yourself that you are not the only one in this situation! Taking steps to start doing more physical activity is no simple task. Many people living with HIV find advantages in physical activity. Being active makes them feel fit, helps them control their weight, and improves flexibility in order to prevent aches, pains and muscular atrophy. It also improves oxygen flow to the brain. Studies have shown that adopting habits such as performing physical activity reduces the risk of heart disease and diabetes. Additionally, it helps unwind and restore your sense of well-being. I invite you to listen to Daniel's story about the advantages and disadvantages of physical activity.

To improve perceived behavioral control, the following methods are deployed: control belief selection, coping planning, and verbal persuasion. Finally, there are two preferred methods for acting on intention: implementation of intention and goal setting.  Table 2 gives the targeted determinants and their corresponding theory-based intervention methods, definitions, parameters for use, and applications/messages. The table is modeled on the one presented in Bartholomew et al. (2011) [24].

### 3.4. Step  4: Content and Sequences of the Intervention

*TAVIE en santé* is a tricomponent web-based tailored intervention facilitated by a virtual nurse. It is meant to address smoking cessation (SC), physical activity (PA), and healthy eating (HE). The virtual nurse is at the heart of each web session. This virtual nurse is in fact a real nurse who acts as a “virtual coach” and interacts with PLHIV asynchronously via the medium of video clips. The role of this virtual nurse is to provide education and to propose strategies to participants to support them in adopting their chosen health behavior. She also provides positive reinforcement and feedback on the significant elements of the previous session. This ensures follow-up prior to each new session by verifying the participant understanding of proposed skills and their application in daily life. Aside from delivering tailored teaching, the virtual nurse also refers to the experiences of other people who have been able to cope with similar situations successfully.

Each component (SC, PA, and HE) includes seven interactive web sessions lasting 5 to 10 minutes. The average duration of each component is 50 minutes. Each web session has a distinct objective. The first session focuses on identifying the advantages and disadvantages of adopting the chosen behavior. The second session aims to help the participant remember the advantages of adopting this behavior. The third and fourth sessions are geared to identifying difficult situations and barriers as well as ways to overcome them. The fifth session is intended to help the participant formulate an action plan to facilitate adopting the behavior, and the sixth session is supposed to reinforce this action plan. The seventh session serves as a booster to consolidate what the participants learned during the intervention.

**Figure 2:**
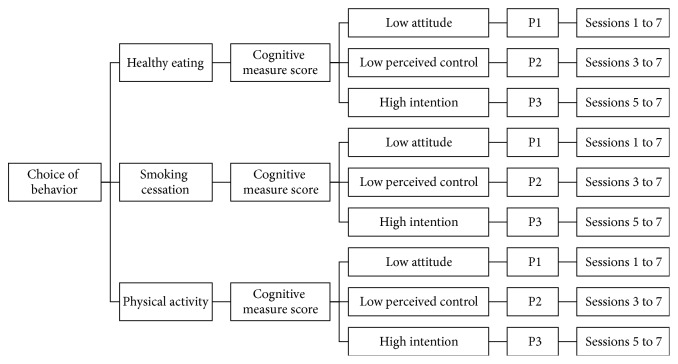
Profile attribution.

The intervention is tailored on level of intention, attitude, and perceived control. Participants are assigned one of three profiles generated by a computer algorithm (see Figure 2). Profile 1 (P1) is marked by low attitude regarding adopting the chosen behavior and requires completing all seven sessions of the intervention. Profile 2 (P2) is characterized by low perceived control and requires completing five sessions (sessions 3 to 7). Profile 3 (P3) corresponds to high intention to change the chosen behavior and requires completing only three sessions (5 to 7). It is assumed that a person with high intention to change (P3) will require less virtual support than a person with low intention to change (P1) or with low perceived control over change (P2). Each profile receives specific, theory-based intervention methods and tailored messages. For example, belief selection and persuasive communication are used to change a participant's attitude towards their chosen behavior. Coping, planning, and role modeling are used to improve perceived behavioral control and goal setting, whereas implementation of intention is used to act on intention.

For the purpose of developing the intervention content (i.e., messages, case stories, scenarios reflecting the reality, and lived experience of PLHIV), a focus group was held with six PLHIV (3 men and 3 women), 30 to 70 years old. The group served to identify behavioral and control beliefs inherent to each behavior. Messages were created in line with focus group content, the theoretical methods used, and the determinants of behaviors chosen.

**Figure 3:**
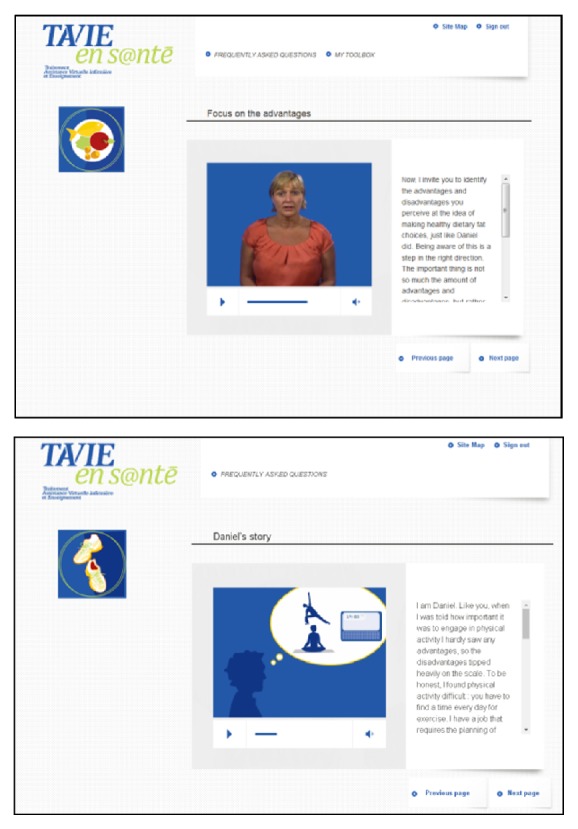
Screenshots of a* TAVIE en santé* web page.

After completing the web sessions corresponding to their profile, participants can revisit all previous sessions as often as they wish via a table of contents. The fact that* TAVIE en santé* is available in both French and English makes it possible to reach a wider audience. The multimedia content of the intervention consists of nearly 350 web pages with 296 videos, 164 animated narrations, and 84 PDF files containing information and tools (see Figure 3).

Our unique team brings together researchers from a variety of disciplines (nursing, medicine, social work, health promotion, and computer science) to pool their expertise in AIDS, cardiovascular disease, and diabetes for a common cause: the development of innovative interventions for the treatment of health problems experienced by PLHIV. Moreover, the content of the intervention has been validated by different health professionals, including a nutritionist, a kinesiologist, nurses, and physical activity research experts.

### 3.5. Steps  5 and 6: Implementation and Evaluation

An online RCT with parallel groups is currently ongoing across Canada. To participate in the study, PLHIV must be ≥18 years old, able to read/understand French or English, and have Internet access. A convenience sample of 750 participants will be assigned randomly either to an experimental group exposed to the* TAVIE en santé* intervention or to a control group with access to informative websites (1 : 1 allocation ratio). Various health behaviors and cognition and health indicators will be assessed at preintervention (T0), 3-month follow-up (T3), and 6-month follow-up (T6). The procedure for this online study will be based on our earlier HIV-Medic Online study [41]. The trial protocol for evaluating the* TAVIE en santé* intervention has been published elsewhere [42].

## 4. Discussion

The purpose of this article was to present results obtained at each step in the development of the* TAVIE en santé* intervention for PLHIV using the IM process and to contribute to the scientific literature on web-based tailored interventions.

There are well-established conceptual frameworks to guide the development of health interventions [24, 43]. The IM method that we used is a systematic approach that draws on theory, empirical evidence, and clinical and experiential knowledge. Identifying the health problem and determinants to target and selecting the most appropriate theoretical methods and practical applications to address these determinants are key steps in the process. Choosing theory-based intervention methods and practical applications suited to the health-related behavior to change and the determinants to target is crucial as well. Michie and colleagues (2008, 2011) have pleaded for the use of theory-based approaches not only to understand behaviors but also to guide intervention design [44, 45]. Unfortunately, interventions are more often theory-inspired than they are theory-based.

For the development of* TAVIE en santé*, IM was chosen over and above other methodologies considering its strengths, added value, and the expertise of the research team associated with this approach. During the development process, some steps were more challenging than others. Steps 1 and 2 were useful in targeting the problem and its determinants. They provided answers to important questions that were prerequisites to the development of the intervention such as the following: what is the goal of the intervention? What are we trying to change? This problem came to light due to a clinical preoccupation to prevent and manage comorbidities among people living with HIV. Three health behaviors were selected: tobacco use, physical inactivity, and poor diet. The current state of empirical and theoretical knowledge facilitated the identification of healthy behavior predictors which became the targeted determinants, namely, intention, attitude, and perceived behavioral control. The challenges arose mainly during steps 3 and 4. We needed to operationalize the content and to find answers to questions such as the following: what strategies for change are available? What and how strategies should be used? The operationalization of the theoretical methods in practical applications and the content development were particularly challenging. During these steps, clinical and experiential knowledge from professionals and patients were highly relevant: they greatly contributed to defining the specific contents and sequences of the intervention. Developing the clinical contents of* TAVIE en santé* required multiple rounds of validation and revisions throughout the process.

While IM allows detailing each step and component of an intervention, it offers little guidance regarding web-based content delivery [46]. In this regard, Morrison and colleagues (2012) stated that existing intervention development approaches needed to be supplemented by frameworks to guide the delivery of content via ICT [47]. Applying critical interpretive synthesis, Morrison and colleagues (2012) proposed four design features that might mediate the effect of ICT interventions on outcomes: self-management, contacts with intervention, tailoring, and social context and support [47]. Our web-based tailored nursing intervention is meant to empower individuals to adopt healthy behaviors. Through asynchronous contacts, the virtual nurse that facilitates the intervention provides tailored coaching and support to this end. The intervention also presents a role model: a virtual patient in a situation similar to the user's, who shares their experience of adopting the proposed skills and strategies. This is the virtual environment that was created to provide a social context and social support.

Health behavior change constitutes a real challenge. In a systematic review of the literature on communication-related behavior change techniques used in face-to-face lifestyle interventions in primary care, Noordman and colleagues (2012) found that 28 of the 50 studies assessed reported significantly favorable health outcomes [48]. But what of tailored interventions of the sort delivered online? Can a web-based intervention alone help change behaviors? In their systematic review of reviews on online prevention aimed at lifestyle behaviors, Kohl and colleagues (2013) found that overall effects of these interventions are positive but the effect sizes appear to be small [49]. In their meta-analysis of web-delivered tailored health behavior change interventions (*n* = 40 RCT), Lustria and colleagues (2013) found that these were most successful when they targeted general populations (*n* = 26) rather than chronically ill patients (*n* = 6, individuals not screened for any particular chronic illness or other disease factors) [15]. Similarly, according to Murray (2012), web-based interventions for people living with chronic conditions remained a challenge and might be less successful [50].

Finally, people living with a chronic health condition often fail to perceive lifestyle behavior changes as an imperative, especially compared with the other health-related challenges they face, such as adherence to medication intake. Consequently, they often lack the readiness and engagement to enact these changes. The issue of engagement in web-based interventions, that is, the fact that not all participants follow or complete online interventions according to plan, is well documented in the literature [51–53]. In their systematic review, for example, Kelders and colleagues (2012) found that only about 50% of participants adhered rigorously to interventions of the sort [52]. Research has sought to identify the characteristics of eHealth intervention nonengagers [54, 55]. In their study, Kelders and colleagues (2011) found that older age and chronic condition predicted nonuse of a web-based intervention to promote healthy eating and exercising behavior [54]. In their systematic review, Kelders and colleagues (2012) proposed various strategies to boost engagement: shorter intervention segments, more dialogue-based support, and interaction with a counselor [52]. They recommended taking these elements into account when designing a web-based intervention. Alkhaldi and colleagues (2016), instead, found that engagement could be bolstered by technology-based strategies such as the use of emails, text messages, and telephone calls [51]. These findings must be interpreted with caution, however, as the field of engagement strategies is still only at a nascent stage.

## 5. Conclusion

In conclusion, the systematic use of IM allowed us to detail all of the steps and components of the intervention following recommendations specific to tailored interventions and the TIDieR checklist [56]. These instruments and guidelines make for better reporting of the content of behavior change interventions, which is essential to help develop new interventions, refine existing ones, and replicate and disseminate them on a larger scale. The use of IM allows avoiding “black box” interventions that, while they may prove effective, are difficult to repeat and explain for lack of implementation data and an understanding of underlying mechanisms.

## Figures and Tables

**Table 1:** Matrix of objectives.

Performance objectives	Intention and behavior determinants and change objectives		
	Attitude	Perceived control	Intention
(PO1) Identify/choose a health behavior to adopt.	(A1) Understand the importance of adopting the health behavior.	(PC1) Assess the capacity to adopt the health behavior.	(I1) Formulate an intention to motivate the adoption of the behavior.
(PO2) Make the decision to adopt a proposed behavior. Engage in a behavior adoption process. Begin adopting the behavior and act accordingly.	(A2) Identify the advantages and disadvantages of adopting the chosen behavior. Identify the positive and negative emotions in adopting the chosen behavior.	(PC2) Identify the barriers and factors that may hinder or facilitate adoption of the chosen behavior. Identify ways of overcoming the barriers and negative emotions and the external resources that may help adopt the chosen behavior.	(I2) Formulate an action plan describing when, where, and how to adopt the chosen behavior. Anticipate/identify/recognize the barriers and find ways to overcome and foresee the difficulties.
(PO3) Act and consolidate the behavior, overcome potential barriers by deploying means and reminding the advantages of adopting the behavior.	(A3) Evaluate adoption of the behavior positively and focus on the advantages of adopting the chosen behavior.	(PC3) Believe in the ability to use the factors and external resources that may facilitate adoption of the behavior. Use these problem-solving strategies.	(I3) Carry out/reinforce the action plan and act on the barriers encountered.

**Table 2:** Theory-based intervention methods and their practical applications [24, 40].

Targeted determinant	Theory-based method	Definition	Parameters for use	Application/Messages
Basic methods for individual change/methods shared by all profiles	Tailoring (trans-theoretical model)	Match the intervention to previously measured individual characteristics	The variables to act on are those related to determinants in the theory of planned behavior and level of intention	The intervention sequence is selected based on the individual profile determined by a preprogrammed algorithm. Users with low attitude (profile 1) must complete 7 sessions. Users with high intention must complete 3 sessions (lower intensity)
	Modeling (social cognitive theory, theories of learning)	Provide a suitable role model to reinforce the target behavior/action	Identification with a role model, coping model	Identification with a role model who successfully identified advantages, overcame difficult situations, and applied the action plan: “*I realized that for me the positive side of exercising was burning energy. If I burnt energy, I would increase the oxygen flow to my brain and would have better concentration at work. Besides, exercise would help my appearance*.” *(Daniel's story) “IF I lack the time to engage in physical activity, THEN I will… Use my bicycle to go visit my daughter. Take my dog for a walk 3 times a day. Take the stairs instead of the elevator in my building.” (Mr. Sullivan's story)*
	Feedback/reinforcement	Provide participants with information on how they are doing with adopting the behavior	Feedback must be personalized and must track the behavior over time	Individualized messages are selected by the computer from a bank of messages depending on the reported determinant. For example, if one participant identifies barriers to stop smoking and another establishes an action plan, the virtual nurse will respond accordingly: *“Well done! Being aware of the obstacles and difficult situations that can prevent you from quitting smoking is an important step towards achieving your goal.” “Excellent! Setting a specific, measurable, achievable, realistic and timely action plan will help you move into action. Remember that you can use the SMART approach at all times to help and motivate you to become more active. Having an action plan increases your chances of success.” *

To change attitude	Belief selection (theory of planned behavior, theory of reasoned action)	Use messages that strengthen positive behavioral beliefs, weaken negative ones, and introduce new beliefs	Requires solid knowledge of behavioral, normative, and control beliefs before selecting which beliefs to intervene on	The virtual nurse asks participants to identify the advantages and disadvantages of adopting the target behavior and to focus on positive beliefs: “*I invite you to identify the advantages and disadvantages that you perceive in regards to exercising. Realizing what motivates you or discourages you is a step in the right direction. The important thing is not so much the number of advantages or disadvantages you find, but rather the weight you assign to each one! You can also discuss this with someone close to you, a friend, a family member or a health professional. It could help with your awareness of the potential benefits*.”
	Persuasive communication (social cognitive theory)	Guide participants in adopting an idea, attitude, or action by using arguments	Messages must be related to the individual's beliefs. They must be meaningful, surprising, repeated, spaced over time, and easy to understand. Requires cognitive skills	The virtual nurse's messages used in the intervention take these parameters into account. In the following case, the user did not identify the advantages of adopting a behavior: *“You know, changing your attitude or perception towards a habit like exercising is a difficult task. But I know you are capable of doing it! For the time being, it seems that the disadvantages of exercising are tipping your scale. Rest assured, you are not alone in this situation. I encourage you to refocus on the advantages of being active. For example, exercise will help you keep in shape and make you feel good about yourself. It can also reduce the risk of heart disease and diabetes as well as contribute to your overall good health.”*

To improve perceived control (or sense of personal efficacy)	Belief selection (theory of planned behavior, theory of reasoned action)	Use messages that strengthen positive control beliefs, weaken negative ones, and introduce new beliefs	Requires solid knowledge of behavioral, normative, and control beliefs before selecting which beliefs to intervene on	The virtual nurse asks participants to identify difficult situations in adopting the behavior as well as favorable factors: *“As you know, quitting smoking is a complex task. Some situations, obstacles or barriers can make quitting difficult. It is therefore important to identify these obstacles so that an immediate solution can be found to overcome them. For example, it could be that cigarettes give you a certain pleasure and you don't want to give that up. It is also possible that you dread the symptoms of nicotine withdrawal. Maybe you are presently under great stress and going through big changes in your life. The important thing is to identify the difficulties, barriers or obstacles that you face in quitting the habit. It will then be possible to find solutions or ways to help…” “There are things that could help or make it easier for you to quit smoking. This is what people living with HIV answered when I asked them the following question: *‘*What could help you stop smoking?*'” * “What would help me stop smoking would be to be supported either by a group, a program, a counsellor, or a health professional with regular follow-ups.” (patient's story)*
	Coping planning (self-regulation theory)	The participant must identify potential barriers and ways to deal with them	Identify risk situations and find solutions	The nurse invites participants to identify the barriers that prevent them from making good choices regarding fat, stopping smoking, or being physically active. She suggests applying the DECIDE process, which is a problem-solving strategy to overcome barriers: Describe the difficult situation/barrier or obstacle, draw up a list of strategies, choose a strategy, imagine yourself using this strategy, decide to go into action, and evaluate the result.
	Verbal persuasion (social cognitive theory)	Use messages that suggest to participants that they have the ability to adopt the behavior	Credible source	The virtual nurse says the following: *“In this session, we saw the importance of finding ways to overcome obstacles preventing you from making healthy choices regarding dietary fat. I invite you to pursue the process and identify the strategies that could help you. Remember that you are capable of doing it!”*

To act on intention	Implementation of intention (theories of goal-directed behavior)	Encourage participants to make an “IF/THEN” plan that will be triggered at the critical moment in order to attain the behavioral objective	Requires a positive intention	The virtual nurse and PLHIV invite the participant to draw up an action plan and give examples (case story). The “IF/THEN” technique is used: The participant identifies an obstacle (IF this happens) and finds a solution by answering: THEN I will do the following. “*IF I am too tired at the end of the day to do my exercises, THEN I will remember that being active gives me energy and reduces my stress.” *
	Goal setting (self-regulation theory)	Lead participants to plan what they need to do (set a goal) if they are to adopt the behavior	Commit to achieving the objective, which may be difficult though within the participant's capabilities	The virtual nurse invites participants to set an achievable behavioral objective (where, when, and how) by using a SMART (specific, measurable, achievable, realistic, and timely) objective. For example, *“In short, this is how a SMART plan of action can be devised: On my way home from work I'll get off the bus before my regular stop so that I can take a 30-minute brisk walk (how), in the park (where) every evening after work, starting next week (when).” *

## References

[1] Johnson L. F., Mossong J., Dorrington R. E. (2013). Life expectancies of South african adults starting antiretroviral treatment: collaborative analysis of cohort studies. *PLoS Medicine*.

[2] Nakagawa F., May M., Phillips A. (2013). Life expectancy living with HIV: recent estimates and future implications. *Current Opinion in Infectious Diseases*.

[3] Friis-Møller N., Weber R., Reiss P. (2003). Cardiovascular disease risk factors in HIV patients—association with antiretroviral therapy. Results from the DAD study. *AIDS*.

[4] Bradbury R. A., Samaras K. (2008). Antiretroviral therapy and the human immunodeficiency virus—improved survival but at what cost?. *Diabetes, Obesity and Metabolism*.

[5] Butt A. A., McGinnis K., Rodriguez-Barradas M. C. (2009). HIV infection and the risk of diabetes mellitus. *AIDS*.

[6] Samaras K. (2009). Prevalence and pathogenesis of diabetes mellitus in HIV-1 infection treated with combined antiretroviral therapy. *Journal of Acquired Immune Deficiency Syndromes*.

[7] Sax P. E. (2010). Assessing risk for cardiovascular disease in patients with human immunodeficiency virus: why it matters. *Circulation*.

[8] Stein J. H., Hsue P. Y. (2012). Inflammation, immune activation, and CVD risk in individuals with HIV infection. *Journal of the American Medical Association*.

[9] Cioe P. A. (2013). Smoking cessation interventions in HIV-infected adults in north america: a literature review. *Journal of Addictive Behaviors Therapy & Rehabilitation*.

[10] Moscou-Jackson G., Commodore-Mensah Y., Farley J., DiGiacomo M. (2014). Smoking-cessation interventions in people living with HIV infection: a systematic review. *Journal of the Association of Nurses in AIDS Care*.

[11] Jaggers J. R., Dudgeon W., Blair S. N. (2013). A home-based exercise intervention to increase physical activity among people living with HIV: study design of a randomized clinical trial. *BMC Public Health*.

[12] Roos R., Myezwa H., Van Aswegen H., Musenge E. (2014). Effects of an education and home-based pedometer walking program on ischemic heart disease risk factors in people infected with HIV: a randomized trial. *Journal of Acquired Immune Deficiency Syndromes*.

[13] Lazzaretti R. K., Kuhmmer R., Sprinz E., Polanczyk C. A., Ribeiro J. P. (2012). Dietary intervention prevents dyslipidemia associated with highly active antiretroviral therapy in human immunodeficiency virus type 1-infected individuals: a randomized trial. *Journal of the American College of Cardiology*.

[14] Samaras K. (2012). The burden of diabetes and hyperlipidemia in treated HIV infection and approaches for cardiometabolic care. *Current HIV/AIDS Reports*.

[15] Lustria M. L. A., Noar S. M., Cortese J., Van Stee S. K., Glueckauf R. L., Lee J. (2013). A meta-analysis of web-delivered tailored health behavior change interventions. *Journal of Health Communication*.

[16] Hou S., Charlery S. R., Roberson K. (2014). Effects of an education and home-based pedometer walking program on ischemic heart disease risk factors in people infected with HIV: a randomized trial. *Health Psychology and Behavioral Medicine*.

[17] Kreuter M. W., Clark E. M., Oswald D. L., Bull F. C. (1999). Understanding how people process health information: a comparison of tailored and nontailored weight-loss materials. *Health Psychology*.

[18] Kreuter M. W., Farrell D., Olevitch L., Brennan L. (1999). *Tailored Health Messages: Customizing Communication with Computer Technology*.

[19] Kreuter M. W., Skinner C. S. (2000). Tailoring: what's in a name?. *Health Education Research*.

[20] Dijkstra A. (2005). Working mechanisms of computer-tailored health education: evidence from smoking cessation. *Health Education Research*.

[21] Dijkstra A., De Vries H. (1999). The development of computer-generated tailored interventions. *Patient Education and Counseling*.

[22] De Vries H., Brug J. (1999). Computer-tailored interventions motivating people to adopt health promoting behaviours: introduction to a new approach. *Patient Education and Counseling*.

[23] Oldenburg B., Taylor C. B., O'Neil A., Cocker F., Cameron L. D. (2015). Using new technologies to improve the prevention and management of chronic conditions in populations. *Annual Review of Public Health*.

[24] Bartholomew L., Parcel G. S., Kok G., Gottlieb N. H., Fernández M. E. (2011). *Planning Health Promotion Programs: An Intervention Mapping Approach*.

[25] Bartholomew L. K., Parcel G. S., Kok G., Gottlieb N. H. (2006). *Planning Health Promotion Programs: An Intervention Mapping Approach*.

[26] Lifson A. R., Lando H. A. (2012). Smoking and HIV: prevalence, health risks, and cessation strategies. *Current HIV/AIDS Reports*.

[27] Mdodo R., Frazier E. L., Dube S. R. (2015). Cigarette smoking prevalence among adults with HIV compared with the general adult population in the United States: cross-sectional surveys. *Annals of Internal Medicine*.

[28] Nahvi S., Cooperman N. A. (2009). Review: the need for smoking cessation among HIV-positive smokers. *AIDS Education and Prevention*.

[29] Tesoriero J. M., Gieryic S. M., Carrascal A., Lavigne H. E. (2010). Smoking among HIV positive New Yorkers: prevalence, frequency, and opportunities for cessation. *AIDS and Behavior*.

[30] Santos-Lozano A., Garatachea N. (2011). Physical activity measurements using accelerometers and pedometers in HIV-infected people. *Journal of AIDS and Clinical Research*.

[31] Schuelter-Trevisol F., Wolff F. H., Alencastro P. R. (2012). Physical activity: do patients infected with HIV practice? How much? A systematic review. *Current HIV Research*.

[32] Klassen K., Goff L. M. (2013). Dietary intakes of HIV-infected adults in urban UK. *European Journal of Clinical Nutrition*.

[33] Giudici K. V., Duran A. C. F. L., Jaime P. C. (2013). Inadequate food intake among adults living with HIV. *São Paulo Medical Journal*.

[34] Joy T., Keogh H. M., Hadigan C. (2007). Dietary fat intake and relationship to serum lipid levels in HIV-infected patients with metabolic abnormalities in the HAART era. *AIDS*.

[35] Godin G. (2012). *Les Comportements dans le Domaine de la Santé: Comprendre Pour Mieux Intervenir*.

[36] Armitage C. J., Conner M. (2001). Efficacy of the theory of planned behaviour: a meta-analytic review. *British Journal of Social Psychology*.

[37] Godin G., Kok G. (1996). The theory of planned behavior: a review of its applications to health- related behaviors. *American Journal of Health Promotion*.

[38] Sheeran P. (2002). Intention—behavior relations: a conceptual and empirical review. *European Review of Social Psychology*.

[39] Webb T. L., Sheeran P. (2006). Does changing behavioral intentions engender behavior change? A meta-analysis of the experimental evidence. *Psychological Bulletin*.

[40] Gagnon H., Côté J., Tessier S., April N. (2012). Développement d’une plateforme Web pour réduire l’usage de cannabis chez les jeunes qui fréquentent les centres d’éducation des adultes. *Drogues, santé et société*.

[41] Côté J., Godin G., Guéhéneuc Y.-G. (2012). Evaluation of a real-time virtual intervention to empower persons living with HIV to use therapy self-management: study protocol for an online randomized controlled trial. *Trials*.

[42] Côté J., Cossette S., Ramirez-Garcia P. (2015). Evaluation of a Web-based tailored intervention (TAVIE en santé) to support people living with HIV in the adoption of health promoting behaviours: an online randomized controlled trial protocol. *BMC Public Health*.

[43] Green L., Kreuter M. (1999). *Health and Promotion Planning: An Educational and Ecological Approach*.

[44] Michie S., van Stralen M. M., West R. (2011). The behaviour change wheel: a new method for characterising and designing behaviour change interventions. *Implementation Science*.

[45] Michie S., Johnston M., Francis J., Hardeman W., Eccles M. (2008). From theory to intervention: mapping theoretically derived behavioural determinants to behaviour change techniques. *Applied Psychology*.

[46] Pellegrini C. A., Steglitz J., Hoffman S. A. (2014). e-Health intervention development: a synopsis and comment on ‘What design features are used in effective e-Health interventions? A review using techniques from critical interpretive synthesis’. *Translational Behavioral Medicine*.

[47] Morrison L. G., Yardley L., Powell J., Michie S. (2012). What design features are used in effective e-health interventions? A review using techniques from Critical Interpretive Synthesis. *Telemedicine Journal and E-health*.

[48] Noordman J., van der Weijden T., van Dulmen S. (2012). Communication-related behavior change techniques used in face-to-face lifestyle interventions in primary care: a systematic review of the literature. *Patient Education and Counseling*.

[49] Kohl L. F. M., Crutzen R., De Vries N. K. (2013). Online prevention aimed at lifestyle behaviors: a systematic review of reviews. *Journal of Medical Internet Research*.

[50] Murray E. (2012). Web-based interventions for behavior change and self-management: potential, pitfalls, and progress. *Journal of Medical Internet Research*.

[51] Alkhaldi G., Hamilton F. L., Lau R., Webster R., Michie S., Murray E. (2016). The Effectiveness of prompts to promote engagement with digital interventions: a systematic review. *Journal of Medical Internet Research*.

[52] Kelders S. M., Kok R. N., Ossebaard H. C., van Gemert-Pijnen J. E. W. C. (2012). Persuasive system design does matter: a systematic review of adherence to web-based interventions. *Journal of Medical Internet Research*.

[53] Reinwand D. A., Crutzen R., Elfeddali I. (2015). Impact of educational level on study attrition and evaluation of web-based computer-tailored interventions: results from seven randomized controlled trials. *Journal of Medical Internet Research*.

[54] Kelders S. M., Van Gemert-Pijnen J. E. W. C., Werkman A., Nijland N., Seydel E. R. (2011). Effectiveness of a web-based intervention aimed at healthy dietary and physical activity behavior: a randomized controlled trial about users and usage. *Journal of Medical Internet Research*.

[55] Schulz D. N., Schneider F., de Vries H., van Osch L. A. D. M., van Nierop P. W. M., Kremers S. P. J. (2012). Program completion of a web-based tailored lifestyle intervention for adults: differences between a sequential and a simultaneous approach. *Journal of Medical Internet Research*.

[56] Hoffmann T. C., Glasziou P. P., Boutron I. (2014). Better reporting of interventions: template for intervention description and replication (TIDieR) checklist and guide. *BMJ*.

